# How mantle heterogeneities drive continental subduction and magmatism in the Apennines

**DOI:** 10.1038/s41598-022-17715-w

**Published:** 2022-08-10

**Authors:** G. Giacomuzzi, P. De Gori, C. Chiarabba

**Affiliations:** ONT Department, Istituto Nazionale di Geofisica and Vulcanologia, 00143 Rome, Italy

**Keywords:** Geodynamics, Seismology, Tectonics

## Abstract

Petrologic and geophysical observations floored the paradigm shift on the subduction of the continental lithosphere. In long-lived collisional boundaries like the Alpine Himalaya belt, portions of continental lithosphere are pushed down to great depths and then exhumed, as testified by outcrops of UHP materials. The Mediterranean region is a clear expression of this enigmatic process. On a short space and time scale, the Apennines exhibits a complex pattern of across-belt extension, associated with under-thrusting of continental lithosphere and a variegated suite of magmatic products. Here we show that the delamination of the crust is essential to favor the subduction of the continental lithosphere, a process that is controlled by pre-existing heterogeneity of the uppermost mantle. Teleseismic tomography revealed significant compositional anomalies in the uppermost mantle that controlled the way in which the lithosphere is delaminated. The continental subduction is associated with magmatism, where the variety of products reflects differences in mantle metasomatism that are only in part related to the subduction process.

## Introduction

Continental subduction occurs in spots along the great Alpine-Himalayan belt and in a few other orogens^1–3^, but many aspects on how the continental lithosphere reaches such great depths are still unclear. Ultrahigh-pressure (UHP) metamorphic rocks are found in many Phanerozoic orogens, evidencing that the continental crust subducts to depths greater than 90–100 km before being exhumed by different mechanisms^4–8^. Intermediate depth seismicity describes clear dipping planes within the continental lithosphere, as in the Pamir–Hindu Kush collision system^9^ and in the Apennines belt^10,11^.

**Figure 1:**
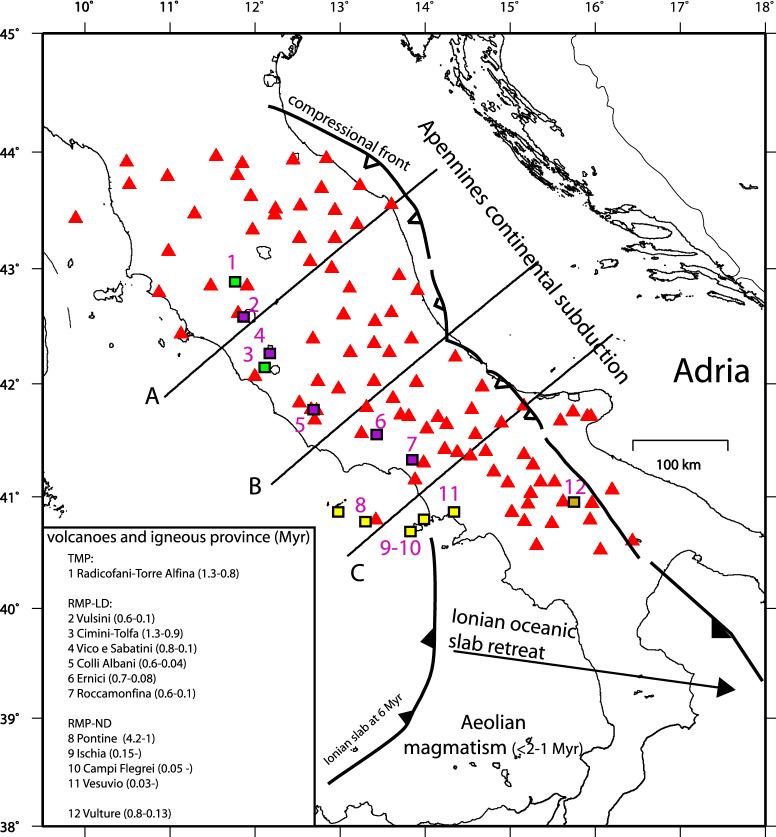
Sketch of the study area showing the seismic stations (red triangles) used in the tomography, main magmatic centers with date or activity, and tectonic lineaments. Classification from^20^ green = Tuscan Magmatic Province (TMP), purple = Roman Magmatic Province Latium district (RMP-LD), yellow = Roman Magmatic Province Neapolitan district (RMP-ND). Traces of vertical sections in Fig. 3 are shown.

The large availability of multidisciplinary data makes the Apennines a strong case study for addressing the open questions on continental subduction. The complexity of such processes is exemplary here, with prolonged subduction that fades in space and time into collision with under-thrusting of the continental lithosphere^12,13^. In this heterogeneous scenario, a surprisingly large range of compositions in the Pliocene–Quaternary igneous records is observed^14,15^ (Fig. 1). It is largely acknowledged that subduction and slab dehydration drive mineral transformations and partial melting of the mantle wedge^16^. The amount of recycled crust and release of fluids and hydrous melts are primary factors influencing the mantle metasomatism and the generation of magma^17^. The great variability includes magmas deriving from an enrichment by dehydration of subducted sediments^18^.

Despite all such information, open questions remain on the continental slab metamorphism and how it affects wedge hydration process, on the depth to which the continental lithosphere might subduct^19^, on the alteration of mantle composition^20^ and connection between magma uprise and belt evolution^21^. Evolutionary models of mountain belts derive from the assumption that high Vp anomalies delineate cold subducting material, being the velocity anomaly in the mantle preferentially related to difference in temperature^22^. Anyway, compositional anomalies in the mantle cannot be individuated considering only Vp models. It is a joint interpretation of Vp and Vp/Vs anomalies that document how the upper mantle might be heterogeneous in composition^23^. Here, we aim to infer insight on the continental subduction and how this connects with the wide suites of magmatism, by computing Vp and Vs models of the Apennines down to 200 km depth. This depth interval (80–200 km), prevalently illuminated by teleseismic data, is crucial for understanding the relation between subduction and magmatism. By combining velocity models with geophysical and petrological information, we propose a model for the structure and composition of the uppermost mantle that links the anomalous magmatism with the evolution of the subduction system.

## Results

**Figure 2:**
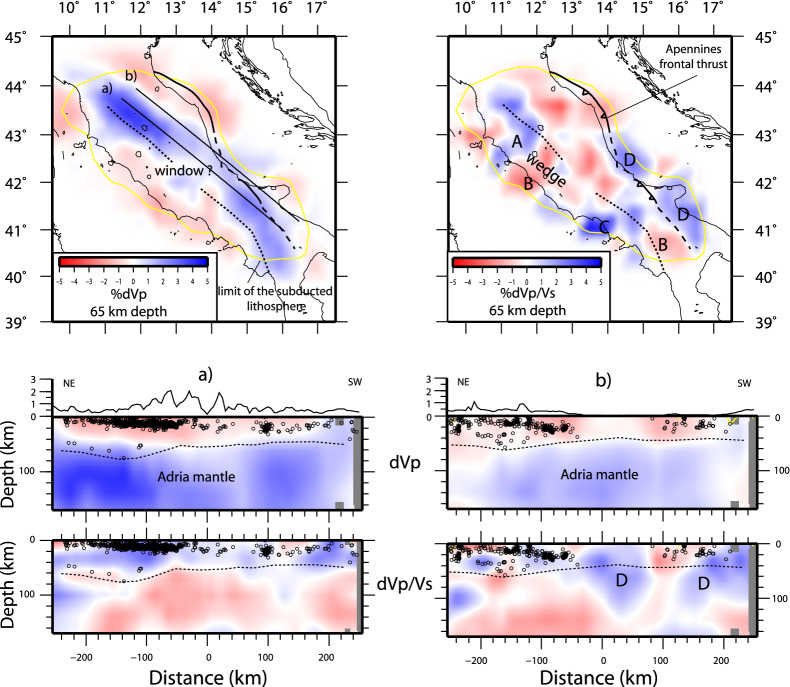
Vp and Vp/Vs models at 65 km depth and in two vertical sections along the Apennines slab. The main anomalies discussed in the paper are indicated by labels. Seismicity is shown in the vertical sections.

**Figure 3:**
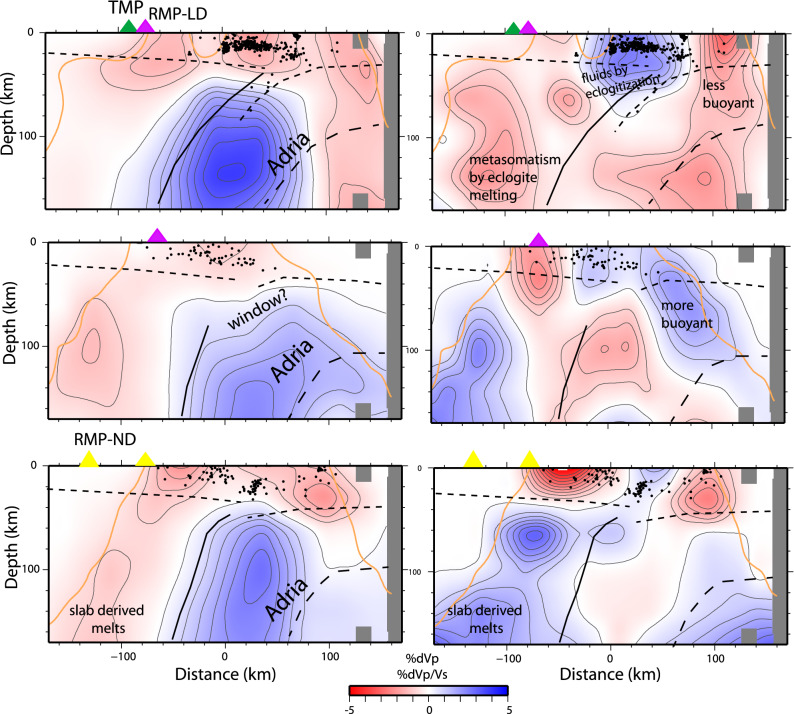
Vertical section of Vp and Vp/Vs in three sections across the Apennines subduction (north, central, south). Dots are seismicity and the geometry of the Moho depth from RFs study^26^ is shown by dashed lines. Triangles are the location of main magmatism. The orange line is the limit of the resolved region according to the SF.

High Vp and low Vp/Vs anomalies define the subducting Adria lithosphere plunging down to the base of the modeled volume at 170 km depth (Figs. 2 and 3). A zone of Vp reduction and low Vp/Vs in the central Apennines is evident. Vp/Vs is strongly heterogeneous with positive anomalies in the Adria lithosphere (label D) and alternation of positive (A and C) and negative (B) anomalies in the mantle wedge. All these anomalies are in well resolved model volumes.

The high Vp, low Vp/Vs subducting Adria continental lithosphere is evident along the belt, with reduced Vp positive anomaly in the central window section, consistent with previous studies^24,25^. In the northern portion (Fig. 3a), the slab anomaly is continuous from 40 km depth to the base of the model, and parallel to a westward dipping plane of seismicity down to 70–80 km depth^11^. Beneath the Central Apennines (Fig. 3b), the Vp decreases between 40 and 100 km depth (from 4–5% to 1–1.5%, Fig. 2), but Vp/Vs is low (i.e., high Vs). The concomitant absence of sub-crustal seismicity matches with a high belt elevation and an almost flat Moho depth^26^, features that motivated a complexity of the subduction^27,28^. Further south (Fig. 3c), the high Vp, low Vp/Vs slab resumes at 40 km depth at the edge of the Ionian oceanic slab.

## Discussion

Subduction models of the Apennines have been elaborated based on geologic data and broad scale P-wave tomography^12,13^. At a closer scale, the subduction panel underwent a progressive disruption^27^ and intermediate depth earthquakes occur only in spots^10^. The cause of such complexity is still unaddressed. Moreover, a subduction-related source for the magmatism along the Apennines is in doubt^29^, and alternative models were proposed for the uprising of melts, including slab tears^30^ or slab windows^31,32^. These latter are ambiguous features introduced to explain the lateral lack of continuity of high P-wave velocity anomalies along a subduction^33^. One of such windows is supposed to exist along the Apennines, in its central/southern sector^24,25^.

In our new model, a broad elongated P-wave velocity anomaly identifies the Adria continental lithosphere beneath the Apennines. This anomaly is consistent with past tomographic studies^24,34^, but its lateral and vertical continuity is strongly enhanced. While the Vp model is rather simple and shows only a central region of reduced anomaly, the Vp/Vs distribution has instead strong lateral heterogeneities. This evidence points to the presence of significant compositional anomalies in the Adria lithosphere and in the Tyrrhenian mantle wedge (Fig. 2). We associate such heterogeneities to a diachronous and irregular process of continental subduction and peculiarity in magmatism.

### Adria mantle anomalies and subduction

**Figure 4:**
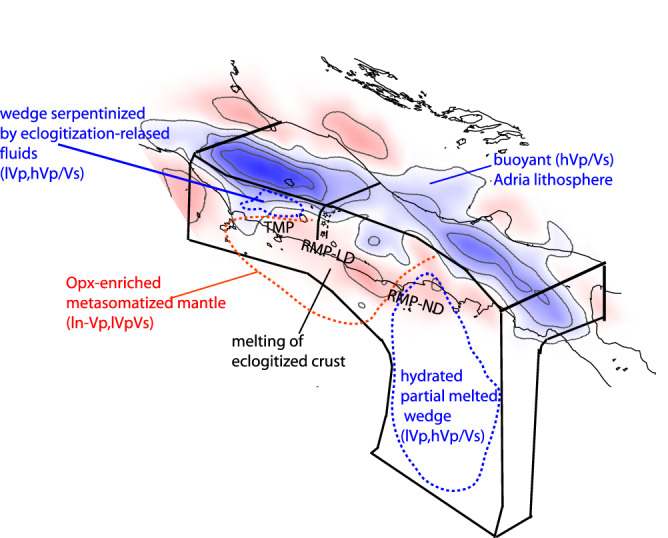
Interpretative sketch of the Apennine subduction, with velocity anomalies at 65 km depth. The main anomalies in the uppermost mantle are reported.

The lateral heterogeneities of the Adria mantle (high and low Vp/Vs) are correlated with differently advancing portions of the compressional front (Figs. 2 and 4, label D). We hypothesize that crustal delamination progressed less efficiently, slowing the lithospheric retreat, when more buoyant portions (high Vp/Vs, label D) of the mantle arrived at the trench. In such a scenario, the attitude of the continental mantle to delaminate controls the subduction retreat, with space/time irregularity that originates from the difference in buoyancy of the anomalous continental mantle. This pre-existing heterogeneity generates portions that progress and retreat differently, forming arcs and recesses of the accretionary front. The delamination, and then the subsequent subduction of the delaminated remnants, can be hampered or even stalled by the arrival at the trench of low velocity, less dense continental mantle portions. We hypothesize that this is what had occurred in the Apennines, progressively halting the process.

We interpret the high Vp/Vs anomalies as hydrated portions of the continental mantle that could be either an inherited feature acquired before the onset of subduction or related to the eclogitization process. The former explanation is consistent with the paleogeography of Adria, with the Mesozoic margin structured in highs and lows, during the diffuse extension that affected the uppermost mantle. The alternative cause is intriguing and dynamically related to a delay in the eclogitization as a consequence of the fast pulling down of the lithosphere driven by the adjacent oceanic slab rollback. A colder geotherm might inhibit the mineralogical transformations, hypothesis consistent with the lack of intermediate-depth earthquakes and the low heat flow of the Adria foreland.

In the central section where the slab window is hypothesized^24,25^, the low Vp/Vs indicates high Vs, not consistent with an asthenosphere upraise through the window. The high Vs suggests a compositional anomaly of the Adria continental mantle, i.e., an Orthopyroxene-enrichment from previous subductions, rather than a physical interruption of the slab. We favor the hypothesis that pre-existing compositional anomalies of the continental lithosphere might explain the reduced Vp and low Vp/Vs alternatively to the slab window. Accordingly, the model of slab tear and segmentation should be revisited, while the continental subduction seems laterally continuous with complexity deriving from the heterogeneity of the untrenched lithosphere. More in general, our results indicate that subduction evolution and complexity in slab geometry like slab windows might be alternatively explainable as compositional anomalies of the subducting continental lithosphere.

### Wedge anomalies and magmatism

Anomalies in the Tyrrhenian mantle wedge varies from high Vp/Vs in northern Apennines (label A) to low Vp, low Vp/Vs (label B) in central Apennines, to high Vp/Vs and low Vp in the southern area (label C). In northern Apennines, high Vp and high Vp/Vs anomalies (label A) are confined at 65 km depth above the dipping seismicity plane. Seismicity occurs for the embrittlement by fluids liberated during the eclogitization of the delaminated crust^35^, a process that seems to develop only down to 80 km depth. Seismicity cut-off suggests that deeper liberation of fluids does not occur. We interpret the anomalies as the wedge serpentinization by fluids released during the eclogitization of the delaminated crust. This is restricted to the mountain belt, as well captured by receiver function studies^19,35^ and not correlated with the magmatism of the Tyrrhenian side. Conversely to oceanic subductions, the fluid liberation from the subducting material seems to be confined at shallow depths, hydrating the wedge but not extending to zones of the mantle where magma is normally generated (< 100 km). If a deeper process occurs is not indicated by seismicity in the subducting lithosphere. The magmatism in the Tyrrhenian area is indeed associated with low Vp/Vs anomalies (B), that support the idea of a metasomatized mantle in absence of an asthenosphere uprise. Si-rich melts derived from melting of the eclogitized continental crust^15,36^ might explain the anomaly, although we cannot clarify if the mantle alteration is coeval with the Adria subduction. The metasomatic signature of the continental mantle might be pre-existing and the retreat of the Adria lithosphere might have favored the decompression and the rise of melts. This hypothesis better agrees with the spatial distribution of magmatism more regular than the Adria delamination along the belt. We interpret this magmatism as related to mantle metasomatism from eclogite melting that sourced magma and products of exotic potassic to ultrapotassic composition^37^.

In the internal Tyrrhenian mantle wedge, a low Vp, high Vp/Vs (label C) volume typical of sediment melts during oceanic subduction is present (Fig. 3c). This melted region was reasonably originated by metamorphism of the Ionian oceanic slab, generated when it was closer to the area (6–4 Myr^27^). The source for the Pontine Island volcanism (which onset was at 4 Myr) was probably related to this melted volume. This anomaly is broadening underneath the southern Tyrrhenian margin and part of the belt also at smaller depths (65 km, Fig. 3), suggesting that also the more recent magmatism of the Neapolitan area can be at least partially sourced by the same melted volume.

Our results support the idea that continental subduction proceeds after the delamination of part of the crust, controlled by lateral heterogeneities in composition of the uppermost mantle. The heterogeneous process of delamination and retreat of the continental lithosphere is conditioned by the entrenching of differently buoyant blocks, giving rise to the compressional belt complexity observed at the surface. Anomalies in the mantle wedge are also distinctive, with low Vp/Vs anomalies associated with mantle metasomatized by Si-rich melts not necessarily coeval with the subduction of delaminated continental material. Conversely, low Vp and high Vp/Vs anomalies mark the wedge volume that is typically altered by subduction-related metasomatism. Oceanic and continental subduction produce different metasomatism of the mantle, with distinctive geophysical signatures. The great variability of magmatic types that characterizes the volcanic suits in the central Mediterranean reflect this heterogeneous metasomatism of the mantle acquired during the long geodynamic history.

## Methods

We use P- and S-wave arrival times from 80 teleseismic events read at 129 seismic stations operating in the Apennines area (Fig. 1). The events cover epicentral distances in the range 35°–100° optimized for back-azimuth and angle of incidence. Arrival times were determined from the vertical and transverse components by means of the Multi-Channel Cross-Correlation technique^38^ adapted to the study area^23^.

Observed arrival times were computed firstly picking the wave onset at a reference station and shifting all waveforms on the base of the differences with the theoretical travel times computed in the IASP91 1D reference velocity model. This first waveform alignment is refined based on the cross-correlation function between each signal and the reference one and picking errors and mean correlation coefficients are computed. To eliminate the contribution of heterogeneities outside the target volume, relative travel time residuals were computed by removing the mean weighted absolute residuals.

A total of 3977 P- and 3671 S-wave relative residuals were inverted by using the non-linear iterative tomographic method^23^. We parameterized the target volume with a 3D grid of nodes and a spacing varying from 30 to 45 km, from the top to the bottom of the model. The forward problem is solved with a ray-tracing algorithm^39^. After the first iteration, we obtained a variance improvement of 64% and 53% and a final variance of 0.24 s and 0.34 s for the P and S models.

The model reliability is computed by means of the spread function, synthetic tests, and the full analysis of the Resolution matrix. This approach is fully consistent with information from synthetic tests^23^ (see the Online Material). The spread function limiting well resolved nodes are shown in maps and sections.

## Supplementary Information

Supplementary Figures.

## Data Availability

Teleseismic waveforms are available on the EIDA web site (http://www.orfeus-eu.org/data/eida/).
