# Supplementary material for: How mantle heterogeneities drive continental subduction and magmatism in the Apennines
Source: Sci Rep. 2022 Aug 10;12:13631. doi: 10.1038/s41598-022-17715-w (PMC9365790; doi:10.1038/s41598-022-17715-w)
Supplement: Supplementary file 1 — Supplementary Figures. [file 41598_2022_17715_MOESM1_ESM.pdf]

Online Material for Mantle heterogeneities, continental delamination, subduction and magmatism: the Apennines case

Giacomuzzi G., P. De Gori, and C. Chiarabba

Istituto Nazionale di Geofisica and Vulcanologia, ONT Department, 00143, Rome, Italy

The material contains two figures that show the syntetic test for model resolution.

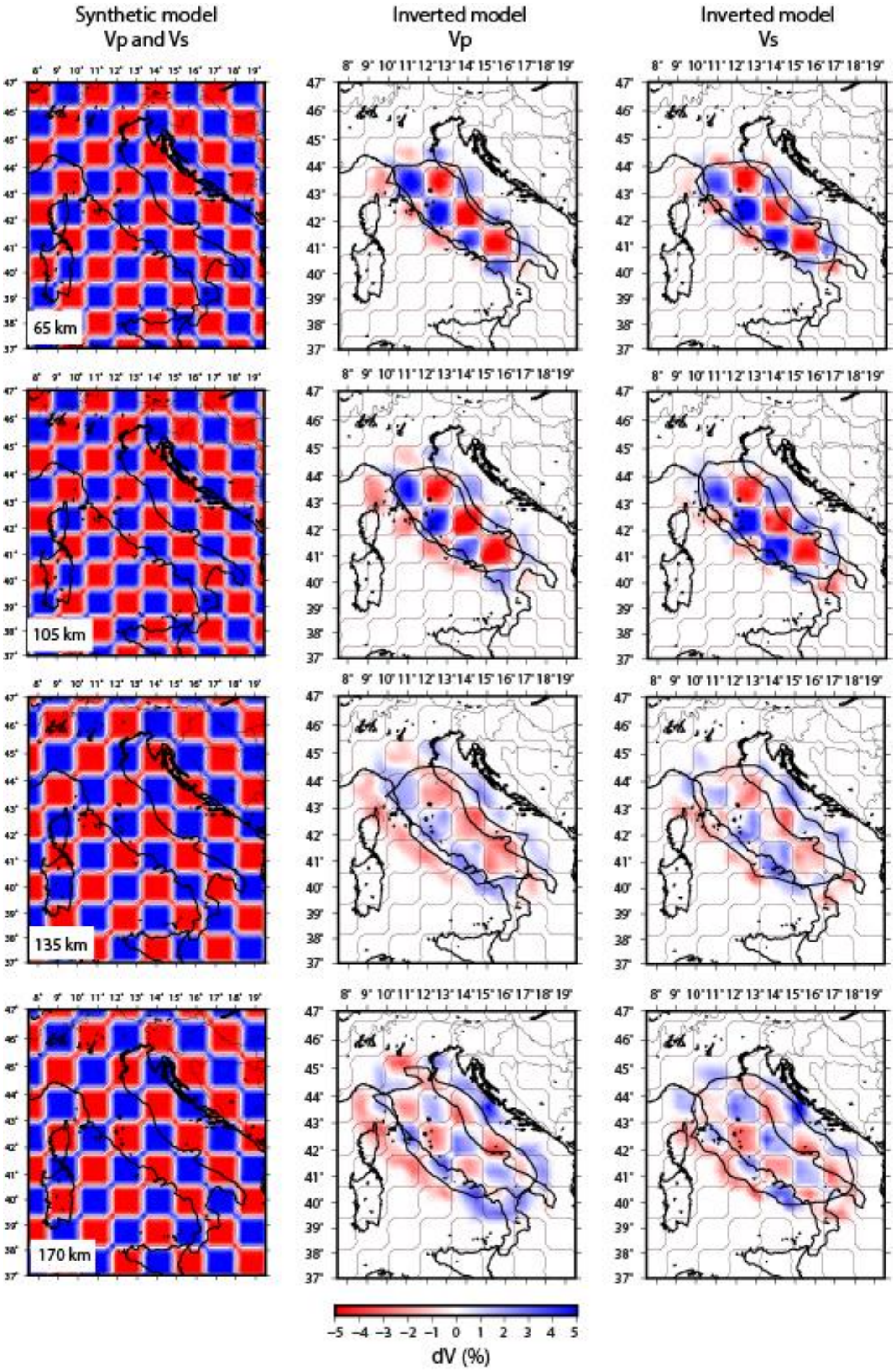

Figure SOM1: Results of the checkerboard test for Vp and Vs models . The reproduction of synthetic anomalies is good in the model, the black lines encircle the resolved areas as defined by the spread function analysis.

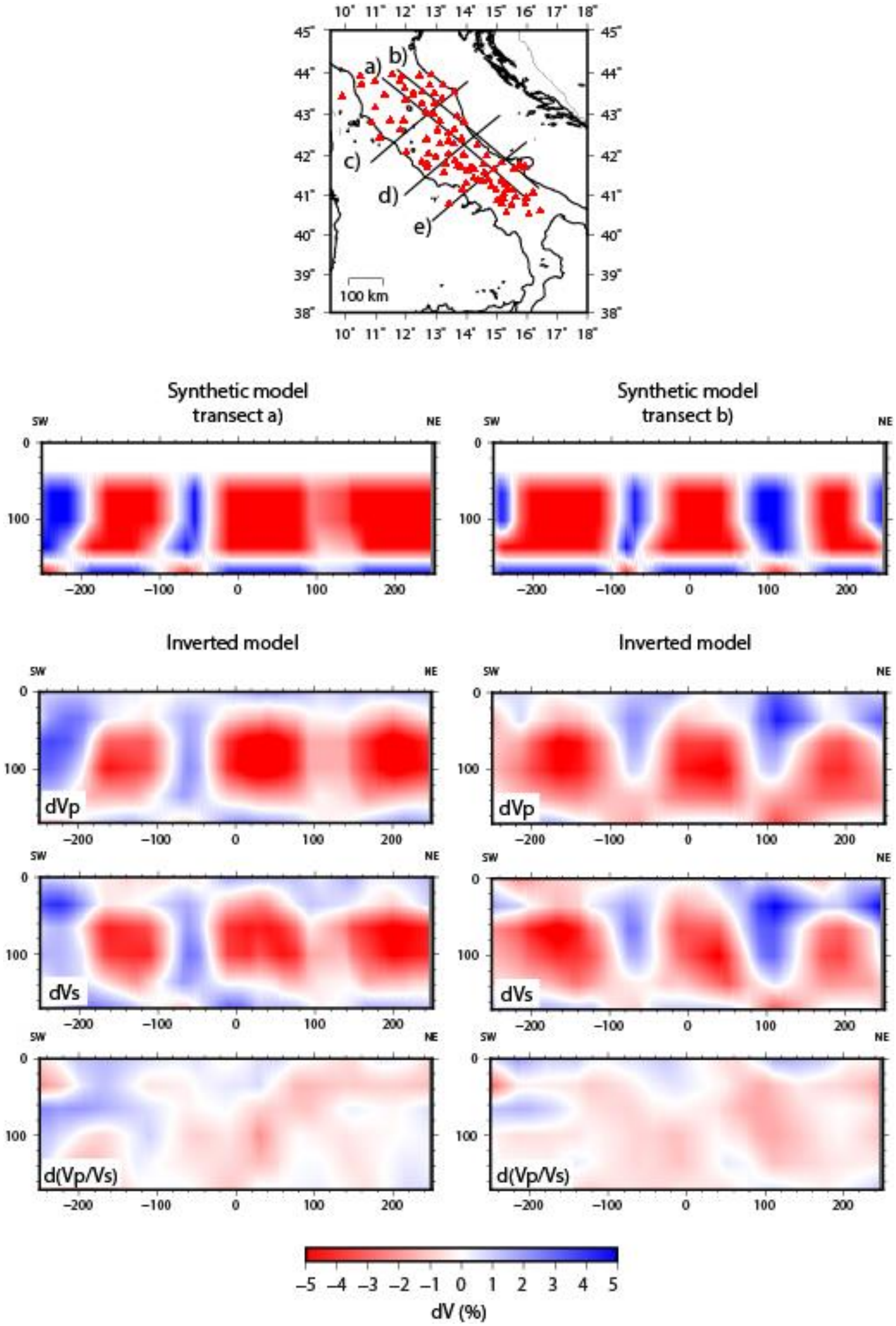

Figure SOM2: Results of the checkerboard test for Vp and Vs models in vertical sections, along the profiles reported in the manuscript.
